# Effects of intranasal dexmedetomidine on postoperative sleep quality: a systematic review and meta-analysis of randomized controlled trials

**DOI:** 10.3389/fmed.2026.1890318

**Published:** 2026-07-10

**Authors:** Jiacheng Zhao, Chao Zhang, Hui Li, Yuxin Qiu, Jinbiao Li, Hui Cao, Xinggen Zhou, Siyao Guan, Jie Wang, Ming Yao

**Affiliations:** 1 Department of Anesthesiology, Suzhou Ninth Hospital Affiliated to Soochow University, Suzhou, China; 2 Department of Anesthesiology, The First Affiliated Hospital, Sun Yat-sen University, Guangzhou, China

**Keywords:** dexmedetomidine, intranasal, meta-analysis, postoperative sleep quality, sleep disturbance, systematic review

## Abstract

**Background:**

Postoperative sleep disturbance is common in surgical patients and may slow recovery. Dexmedetomidine has a sleep like sedative effect, and intranasal administration provides a convenient and non-invasive route. The aim of our study was to evaluate the effect of intranasal dexmedetomidine on postoperative sleep quality in adult surgical patients.

**Methods:**

We performed a systematic review and meta-analysis of randomized controlled trials. PubMed, the Cochrane Library and Embase were searched to May 20, 2026. Intranasal dexmedetomidine was compared with placebo or other controls. Subjective and objective sleep outcomes, sleep disturbance and adverse events were analysed using standard meta-analytic methods. Subgroup analyses were further performed according to the route, timing, and duration of administration, as well as patient characteristics, surgical type, anesthesia type, and evaluation scales.

**Results:**

15 trials were included. For subjective scores where a lower value means better sleep, intranasal dexmedetomidine improved postoperative sleep compared with controls (SMD = −1.29, 95% CI [−1.80, −0.77], *I*^2^ = 90%, *p* < 0.00001). Subgroup analyses suggested that the timing of administration, sex distribution, and surgical type might be important sources of heterogeneity. For scores where a higher value means better sleep, postoperative scores were also higher in the dexmedetomidine group (SMD = 1.75, 95% CI [0.94, 2.57], *I*^2^ = 94%, *p* < 0.0001). Sleep efficiency (SMD = 1.71, 95% CI [0.94, 2.48], *I*^2^ = 93%, p < 0.0001) and total sleep time (SMD = 1.76, 95% CI [0.67, 2.84], *I*^2^ = 91%, *p* = 0.002) increased, sleep latency (SMD = −1.07, 95% CI [−1.66, −0.49], *I*^2^ = 86%, *p* = 0.0003) reduced, while REM sleep, N1 sleep, and N3 sleep did not change. The risk of sleep disturbance decreased (RR = 0.53, 95% CI [0.39, 0.73], *I*^2^ = 74%, *p* < 0.0001). Adverse events such as delirium, nausea and vomiting, and anxiety were less common in the dexmedetomidine group.

**Conclusion:**

Intranasal dexmedetomidine may improve short-term postoperative sleep, but evidence is limited and more high-quality trials are needed.

**Systematic review registration:**

PROSPERO 2025 CRD420251186146, Available from https://www.crd.york.ac.uk/PROSPERO/view/CRD420251186146.

## 1 Introduction

Postoperative sleep problems frequently occur among individuals undergoing surgery and have important clinical effects. Many studies report that between 15 and 70 percent of patients experience sleep problems after surgery under general anesthesia, often with reduced rapid eye movement (REM) sleep, increased fragmentation and disruption of the normal day night rhythm (1). These sleep problems are related to many factors, including surgical stress, pain, high doses of opioids, anxiety and the ward environment such as noise and light (2). Poor perioperative sleep is associated with higher pain scores, slower recovery, more complications and a lower quality of life, and it might also increase the risk of postoperative delirium and cardiovascular events (3). Therefore, maintaining and improving sleep quality after surgery is an important goal in perioperative care and aligns closely with the concept of enhanced recovery pathways.

Dexmedetomidine is an alpha-2 adrenergic agonist with selective activity that produces sedative effects, offers analgesic benefits and helps reduce anxiety (4). It produces an electroencephalogram pattern that is close to non-rapid eye movement (NREM) sleep, and several studies display that dexmedetomidine induced sedation mimics natural stage 3 NREM sleep (5). Clinical studies in surgical patients report that perioperative dexmedetomidine improves postoperative sleep quality, leading to higher sleep efficiency and longer total sleep duration (6). It also appears to lessen light sleep and arousals, although its effect on deep sleep stages and REM sleep is not always consistent (7, 8). Systematic reviews and meta-analyses also find that dexmedetomidine improves postoperative sleep structure and subjective sleep scores when compared with saline (9). In addition, recent reviews from anesthesiology suggest that dexmedetomidine helps to counteract perioperative sleep disturbance and may reduce related complications such as postoperative delirium (10, 11). Overall, the available evidence suggests that dexmedetomidine plays a valuable role in perioperative sleep management and may help patients achieve better sleep during recovery.

Intranasal administration offers a non-invasive and convenient route for dexmedetomidine delivery. Pharmacokinetic studies show that intranasal dexmedetomidine has good bioavailability and provides effective sedation with a relatively rapid onset, both when it is given as drops and as a nasal spray (12). Clinical trials in adults and older patients indicate that intranasal dexmedetomidine can reduce preoperative anxiety (13), provide satisfactory sedation (14) and improve perioperative sleep quality, without major safety problems (15). Recent randomized studies also report that dexmedetomidine nasal spray improves sleep on the first postoperative night after nasal endoscopic surgery (16), maxillofacial surgery (17) and laparoscopic gynecological surgery (18). However, most of these trials have small sample sizes and different doses, timing schedules and sleep assessment tools. At present, no comprehensive systematic review or meta-analysis has been conducted that examines the effects of intranasal dexmedetomidine on postoperative sleep quality in adult surgical patients, so the overall effect and safety profile remain unclear.

Although intranasal dexmedetomidine shows promising effects on perioperative sedation, the evidence for its influence on postoperative sleep quality in adults is still limited and fragmented. The existing trials use different doses, administration methods and sleep evaluation tools, and the results are not always consistent. In addition, no systematic review and meta-analysis has been performed to summarize the current evidence. Therefore, it is necessary to analyse the available clinical studies to clarify the impact of intranasal dexmedetomidine on both subjective and objective postoperative sleep outcomes, as well as its safety profile and influence on sleep disturbance. The purpose of this study is to provide a thorough evaluation of intranasal dexmedetomidine for improving postoperative sleep quality in adult undergoing surgery.

## 2 Methods

This systematic review and meta-analysis adhered to the 2020 Preferred Reporting Items for Systematic Reviews and Meta-Analyses (PRISMA) statement guidelines (19). The PRISMA 2020 checklist was provided in Supplementary Table S1. The study protocol was registered in advance on the International Prospective Register of Systematic Reviews. The registration number was CRD420251186146, and the registration date was 7 November 2025.

### 2.1 Eligibility criteria

Studies were selected based on the Population, Intervention, Comparison and Outcomes framework: (1) Population: Adult patients who received elective surgery; (2) Intervention: Intranasal dexmedetomidine used during the perioperative period. The intranasal drug could be given by drops, spray, or nasal packing; (3) Comparison: Placebo such as normal saline, or dexmedetomidine or other drugs given by another route; (4) Outcomes: Postoperative sleep quality assessed by subjective scores or objective tools; (5) Study design: Randomized controlled trials.

We excluded trials on children, studies that used dexmedetomidine only by intravenous or other non-nasal routes, studies that did not report sleep related outcomes, studies without a control group, and studies that were not randomized controlled trials. We also excluded reviews, conference abstracts without full data, observational studies, animal studies, and studies that were not published in full text.

### 2.2 Search strategy

Two researchers (JCZ and CZ) systematically searched PubMed, the Cochrane Library, and Embase from their inception to May 20, 2026. The search used both subject terms and free text terms, and the main terms were “Dexmedetomidine,” “Administration, Intranasal,” “Sleep,” and “Randomized Controlled Trial.” The complete search methods were listed in Supplementary Table S2, which provided the detailed literature search strategy. We further examined the reference lists of the included articles and relevant review papers to locate any other studies that met the eligibility criteria. The abstracts and titles were checked by them separately for preliminary screening. Studies that met the initial screening criteria were then examined in full text for a more detailed eligibility assessment.

### 2.3 Data extraction

Two reviewers (JCZ and HL) independently extracted data using a predesigned and standardized form. We extracted four groups of data from each study. First, we collected the basic study information, including the first author, publication year, country, age, sex, study population, whether the population had sleep disorders, subjective sleep quality scores, and objective sleep measurements. The subjective scales included the Athens Insomnia Scale (AIS) (20), the Numerical Rating Scale (NRS), the Pittsburgh Sleep Quality Index (PSQI) (21), the Insomnia Severity Index (ISI) (22), St. Mary’s Hospital Sleep Questionnaire (SMHSQ), the Leeds Sleep Evaluation Questionnaire (LSEQ) (23), the Richards Campbell Sleep Questionnaire (RCSQ) (24) and the Subjective Sleep Quality Value (SSQV). The objective sleep data recorded by different devices included sleep efficiency (SE), total sleep time (TST), sleep latency (SL), rapid eye movement (REM) sleep, and sleep stage parameters such as N1, N2, and N3 sleep. Second, we recorded the details of the intranasal dexmedetomidine intervention, such as the method of intranasal use by drops, spray or nasal packing, the dose used for each nostril, the timing of administration, the total dose, the duration, and the type of control group. Third, we extracted the information needed for the risk of bias assessment. Fourth, we recorded the outcome data for the meta-analysis, which included sleep quality, the incidence of sleep disturbance, and different adverse events. If a study only presented data in figures and the numerical values could not be obtained, we contacted the corresponding author or the first author for clarification. If the data were still not available, the study was included only in the systematic review and not in the meta-analysis. Any differences in judgment between the two reviewers were resolved through discussion, and when needed, a third researcher was consulted to reach agreement (YXQ).

### 2.4 Risk of bias and GRADE assessment

Two independent reviewers (JBL and HC) evaluated the methodological quality of the included randomized controlled trials using the Cochrane Collaboration’s Risk of Bias assessment tool. The following seven domains were evaluated: random sequence generation, allocation concealment, blinding of participants and personnel, blinding of outcome assessment, incomplete outcome data, selective reporting, and other potential sources of bias. Each domain was judged as having a low, high, or unclear risk of bias. Discrepancies in assessments were resolved through discussion or, if necessary, by consultation with a third reviewer (XGZ). A summary of the risk of bias evaluation was presented in both tabular and graphical formats. The certainty of evidence for key outcomes was assessed using the Grading of Recommendations Assessment, Development and Evaluation (GRADE) approach. Evidence certainty was rated as high, moderate, low, or very low based on risk of bias, inconsistency, indirectness, imprecision, and publication bias.

### 2.5 Statistical analysis

All statistical analyses were performed using Review Manager (RevMan) version 5.3 and Stata version 17.0. For continuous outcomes, results were expressed as standardized mean differences (SMDs) with 95% confidence intervals (CIs), in order to account for potential variations in measurement units across studies. For dichotomous outcomes, risk ratios (RRs) with corresponding 95% CIs were calculated. When continuous data were presented as median with interquartile range (IQR) or range, the values were converted to mean ± standard deviation (SD) using established methods proposed by Luo et al. (25) and Wan et al. (26). Between-study heterogeneity was assessed using the Cochrane *Q* test and *I*^2^ statistic. An *I*^2^ value > 50% or a *p*-value < 0.10 for the *Q* test was considered indicative of substantial heterogeneity, in which case a random-effects model was applied; otherwise, a fixed-effects model was used. Sensitivity analyses were conducted by sequentially omitting each study to assess the robustness of pooled results. Publication bias was evaluated through funnel plot inspection and Egger’s regression test when at least 10 studies were included in the analysis. A two-tailed *p*-value < 0.05 was considered statistically significant.

For multi-arm trials in which more than one eligible intranasal dexmedetomidine group shared the same control group, we retained the separate dose groups to preserve dose-specific comparisons. To avoid double-counting of participants in the shared control group, the sample size of the control group was divided approximately equally across the relevant comparisons, according to the recommendations of the Cochrane Handbook. When a study included more than one comparator group, the saline group was selected as the control group for the primary meta-analysis whenever available.

## 3 Results

### 3.1 Study selection

**Figure 1:**
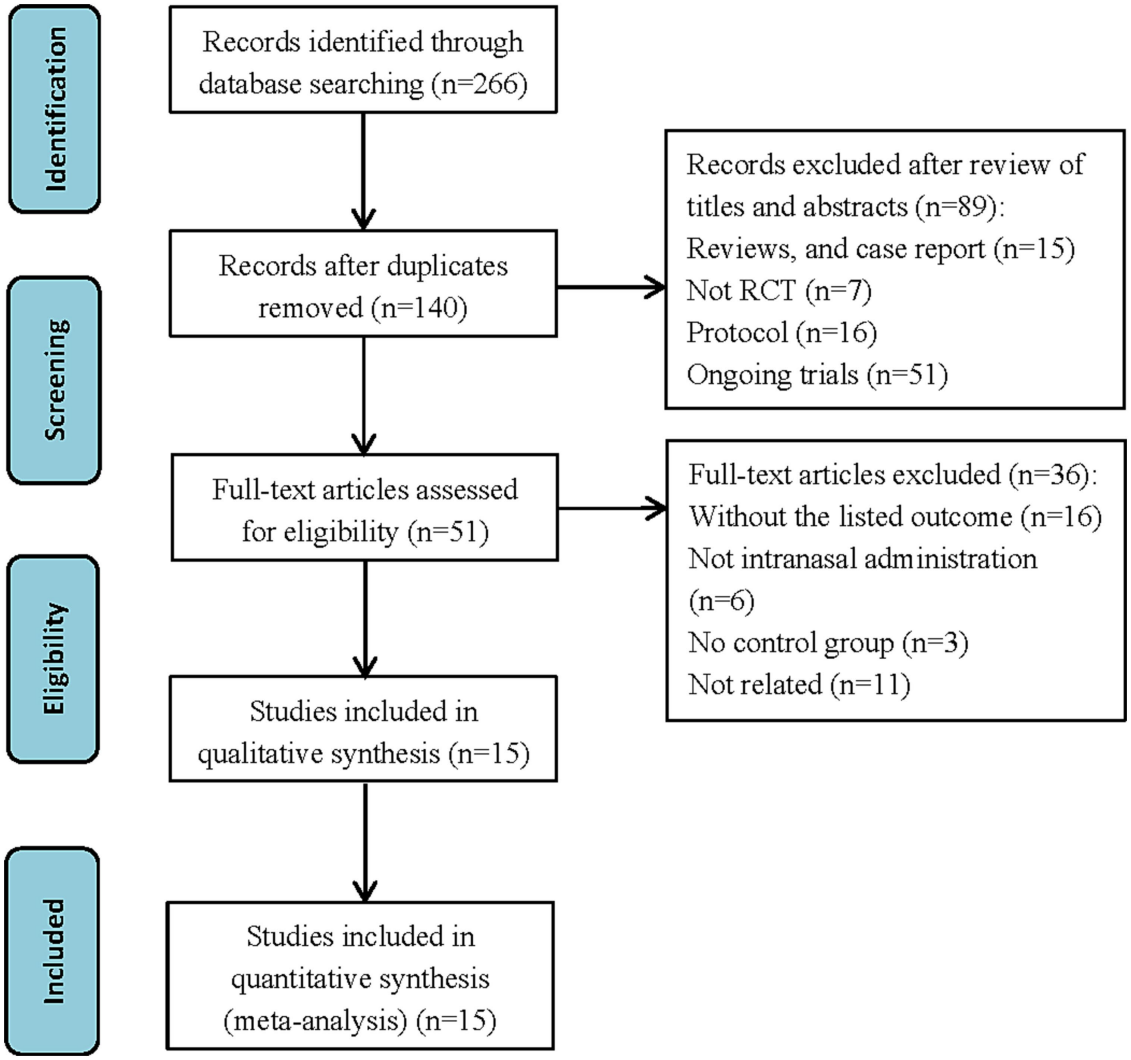
PRISMA flow diagram of study selection.

We identified 266 records through the database search, and 140 records remained after removing duplicates. We screened the titles and abstracts and excluded 89 records. These records were excluded because they were reviews or case reports (*n* = 15), were not randomized controlled trials (*n* = 7), were study protocols (*n* = 16), or were ongoing trials (*n* = 51). We then assessed 51 full text articles for eligibility. Among them, 36 articles were excluded because they did not report the required outcomes (*n* = 16), did not use intranasal administration (*n* = 6), did not include a control group (*n* = 3), or were not related to our topic (*n* = 11). Finally, 15 studies met the criteria and were included in both the qualitative synthesis and the meta-analysis (Figure 1).

### 3.2 Characteristics of the included studies

The characteristics of the included studies, published from 2021 to 2026, are shown in Table 1. All studies were conducted in China and involved adult patients undergoing different elective surgeries or perioperative conditions. Eleven studies used general anesthesia–based techniques, including general anesthesia alone or combined with nerve block anesthesia, while the remaining studies used local infiltration anesthesia, combined spinal-epidural anesthesia, and intravenous anesthesia. The sample sizes of the dexmedetomidine groups ranged from 21 to 174 patients, and the control groups ranged from 18 to 174 patients. The mean ages ranged from about 25 years to about 71 years, and the median ages in some studies ranged from 18 to 77 years. The percentage of male patients also differed considerably among studies, ranging from 0 to 81%. The subjective sleep assessment tools used in the studies included the AIS, NRS, PSQI, ISI, LSEQ, RCSQ, SSQV, and SMHSQ. In addition, 8 studies reported objective sleep data.

**Table 1:** Main characteristics of included studies.

Author, year	Country	Sample size (D/C)	Age (D/C)	Male (D/C)	Participants	Type of anesthesia	Subjective sleep quality assessment	Objective sleep quality assessment
Ling Z, 2026 (34)	China	39/39/39	57.87 ± 4.85/56.56 ± 4.47/58.08 ± 5.34	16 (41%)/15 (38.5%)/13 (33.3%)	Thoracoscopic lung surgery	GA + NBA	AIS, NRS, PSQI, PSD (NRS ≥ 6 or AIS ≥ 6)	/
Tong J, 2026 (35)	China	41/41	61 (50,63)/60 (56,66)	20 (48.8%)/23 (56.1%)	Venous port implantation	LIA	SMHSQ	/
Huang Z, 2026 (36)	China	22/22	62.7 ± 4.6/62.9 ± 5.1	9 (40.9%)/10 (45.5%)	Total knee arthroplasty	CSEA	PSQI	SE, ST, SL, REM, N1, N1, N3
Wang L, 2026 (37)	China	95/94/95	37 (34–46)/39 (35–47)/36 (33–46)	0/0/0	Hysteroscopy	IVA	PSD (PSQI ≥ 15)	ST, REM
Chen C, 2025 (38)	China	174/174	66 (62–72)/67 (63–72)	78 (44.8%)/79 (45.4%)	Major noncardiac surgery	GA	RCSQ	SE, ST, SL, REM
Fan K, 2025 (18)	China	72/72	33.8 ± 0.8/33.4 ± 0.7	0/0	Laparoscopic gynecological surgery	GA	AIS, NRS	SE, ST, REM
Wang D, 2025 (27)	China	80/79	48 (36–60[21–74])/51 (36–61[18–77])	45 (56%)/45 (56%)	Ear, nose and throat surgery	GA	PSD (AIS ≥ 6)	/
Fang J, 2024 (28)	China	50/50	66.9 (3.4)/66.9 (3.4)	31 (62.0%)/28 (56.0%)	Cardiac surgery	GA	PSD (ISI ≥ 15)	/
Yang W, 2024 (29)	China	30/30	50.1 (5.7)/51.3 (4.7)	13 (43%)/10 (33%)	Elective surgery	NR	LSEQ	SE, ST, SL
He J, 2024 (30)	China	40/40	68.45 ± 2.8/69.20 ± 3.01	0/0	Laparoscopic gynecological surgery	GA	RCSQ	/
Niu J, 2023 (31)	China	50/49	70.1 (6.4)/70.8 (6.6)	25 (50.0%)/22 (44.9%)	Spinal surgery	GA	PSQI	/
Wang Y, 2023 (17)	China	39/39/39	25.8 ± 4.9/25.2 ± 5.0/25.7 ± 5.4	4 (10.3%)/4 (10.3%)/8 (20.5%)	Maxillofacial surgery	GA	PSQI	SE, REM, N1, N2, N3
Wu J, 2023 (32)	China	55/55	69 (67,74)/70 (67,73)	22 (40%)/19 (34.5%)	Total hip/knee arthroplasty	GA + NBA	LSEQ, PSQI, ISI	SE, ST, SL
Zeng W, 2022 (33)	China	36/36	38.11 ± 11.47/40.47 ± 11.17	15 (41.7%)/14 (38.9%)	Insomnia and anxiety	NR	ISI	ST
Wang Y, 2021 (16)	China	21/20/21/18	33 (22,46)/33 (22,44)/29 (24,40)/41 (28,61)	17 (81%)/12 (60%)/12 (57%)/13 (72%)	Nasal endoscopic surgery	GA	PSQI, SSQV	/

D, dexmedetomidine; C, control; GA, general anesthesia; NBA, nerve block anesthesia; LIA, local infiltration anesthesia; CSEA, combined spinal-epidural anesthesia; IVA, intravenous anesthesia; AIS, Athens Insomnia Scale; NRS, Numerical Rating Scale; PSQI, Pittsburgh Sleep Quality Index; PSD, postoperative sleep disturbance; SMHSQ, St. Mary’s Hospital Sleep Questionnaire; ISI, Insomnia Severity Index; LSEQ, Leeds Sleep Evaluation Questionnaire; RCSQ, Richards-Campbell Sleep Questionnaire; SSQV, Subjective Sleep Quality Value; SE, sleep efficiency; ST, sleep time; SL, sleep latency; REM, rapid eyes movement; NR, not reported.

### 3.3 Characteristics of intervention

The detailed characteristics of each intervention are shown in Table 2. The included studies used different intranasal methods to give dexmedetomidine, including nasal spray, nasal drip, and nasal packing. Most studies applied the drug by a fixed volume or a fixed number of sprays in each nostril. The timing of administration varied among studies. Ten studies gave the drug before bedtime, whereas 4 studies administered it 20 to 30 min before anesthesia induction. One study administered dexmedetomidine at the end of surgery. The dosage also differed across studies. The fixed doses ranged from 45 to 100 μg, and the weight-based doses ranged from 0.3 to 4 μg/kg. In 11 studies, the drug was given once, while 4 studies used repeated administration for several days before or after surgery. The control groups included saline, blank control, lorazepam, alprazolam, and dexmedetomidine given by intravenous or intratracheal routes.

**Table 2:** Characteristics of administration of intranasal dexmedetomidine.

Author, year	Route of administration	Method of application	Time of intervention	Dosage	Duration	Control group
Ling Z, 2026 (34)	Nasal spray	NR	21:00 to 21:30	1/2 μg/kg	From the night before to 1 day after surgery	Placebo
Tong J, 2026 (35)	Nasal spray	Four sprays	30 min before induction	100 μg	Once	0.9% saline
Huang Z, 2026 (36)	Nasal spray	NR	21:30 on the day of surgery	1 μg/kg	Once	Saline/Intravenous DEX (0.6 μg/kg)
Wang L, 2026 (37)	Nasal drip	0.6 mL/time in each nostril	25–30 min before anesthesia	0.2/0.5 μg/kg	Once	Saline
Chen C, 2025 (38)	Nasal spray	3–5 sprays	20:30 to 00:00	45/60/75 μg	Once	Placebo
Fan K, 2025 (18)	Nasal spray	2 sprays (1 spray in each nostril)	30 min before sleep on the first postoperative night	50 μg	Once	Saline
Wang D, 2025 (27)	Nasal spray	3–4 sprays	30 min before induction	75–100 μg	Once	0.9% saline
Fang J, 2024 (28)	Nasal drip	NR	Before bedtime	0.3 μg/kg	6–8 days before the surgery	Saline
Yang W, 2024 (29)	Nasal drip	0.1 mL/time in each nostril	Before bedtime on the night before surgery	2.5 μg/kg	Once	Saline/lorazepam
He J, 2024 (30)	Nasal drip	0.2 mL/time in each nostril	21:00 to 21:30 every night	1.5 μg/kg	From one day before to 5 days after surgery	Saline/alprazolam
Niu J, 2023 (31)	Nasal drip	0.5 mL/time in each nostril	30 min before anesthesia induction	1.0 μg/kg	Once	Intravenous/intratracheal DEX (0.6 μg/kg)
Wang Y, 2023 (17)	Nasal spray	A small amount of the drug in each nostril	21:30 on the night of the operation	1.0/1.5 μg/kg	Once	Blank control
Wu J, 2023 (32)	Nasal spray	0.1 mL/time in each nostril	21:00 to 21:30	2 μg/kg	From the night after surgery to discharge	Saline
Zeng W, 2022 (33)	Nasal drip	0.1 ml/time in each nostril	Before bedtime	2.5 μg/kg	Once	Saline
Wang Y, 2021 (16)	Nasal packing	NR	At the end of the surgery	1/2/4 μg/kg	Once	Saline

DEX, dexmedetomidine; NR, not reported.

### 3.4 Risk of bias and publication bias assessment

**Figure 2:**
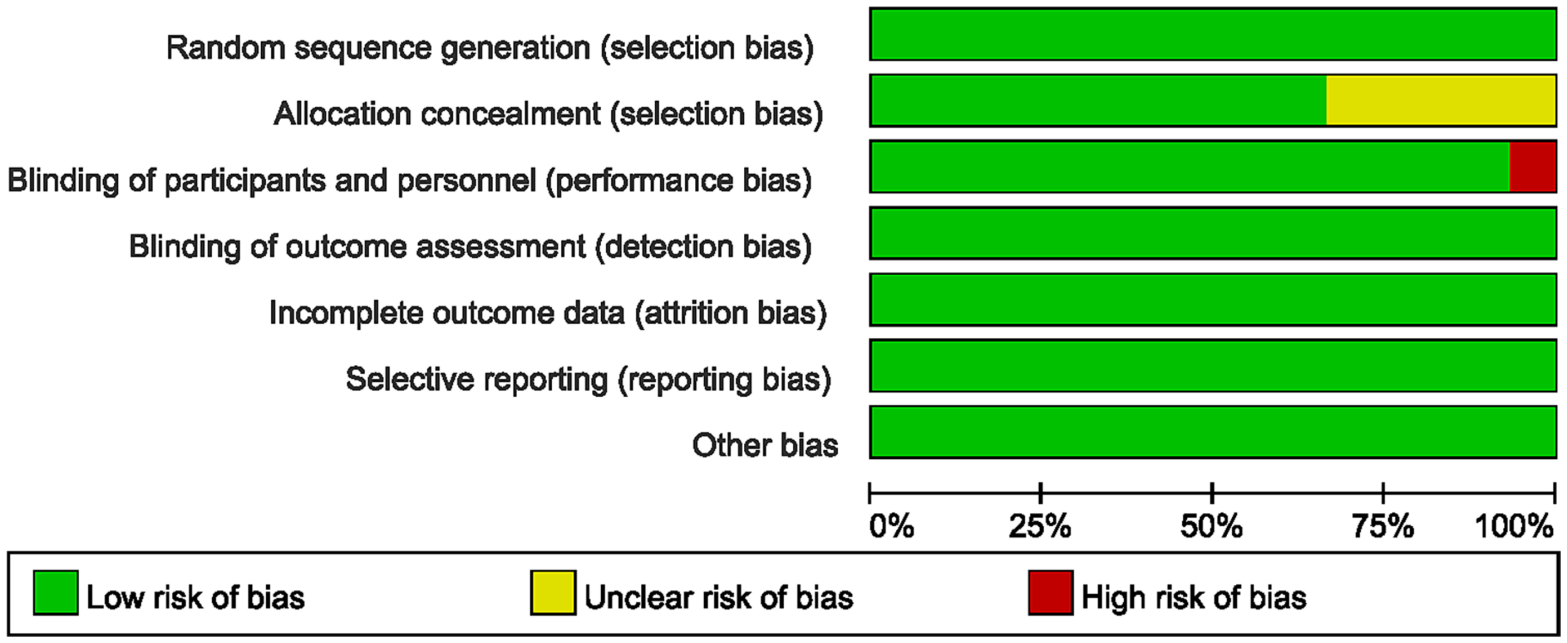
Risk of bias graph.

**Figure 3:**
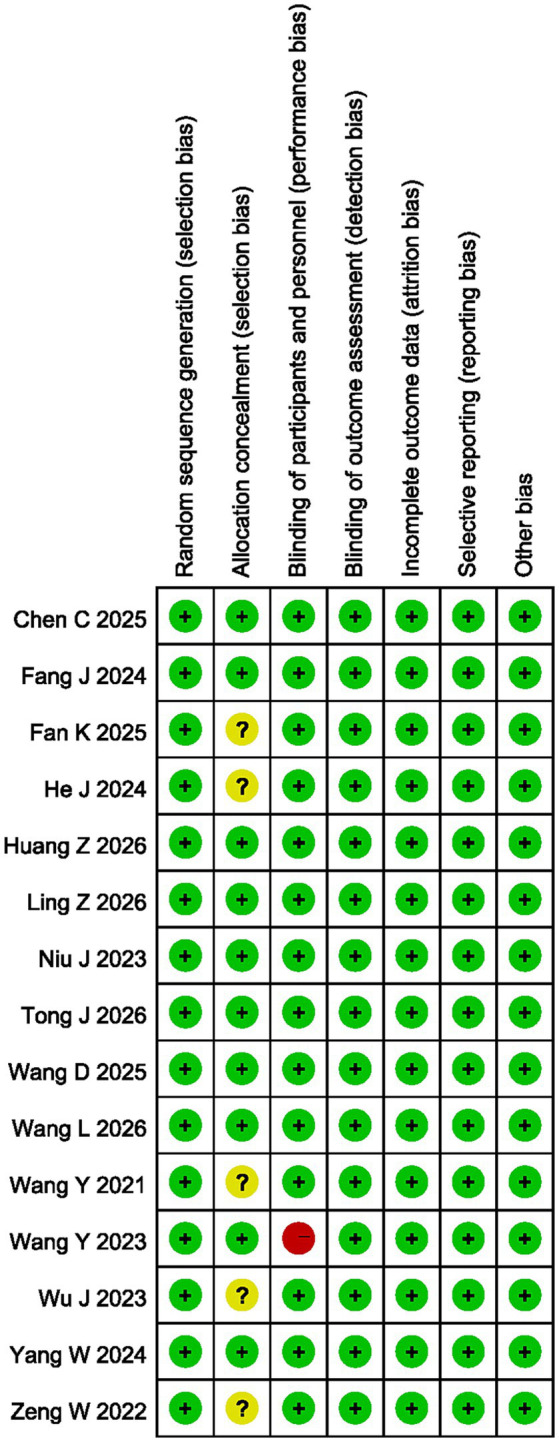
Risk of bias summary.

**Figure 4:**
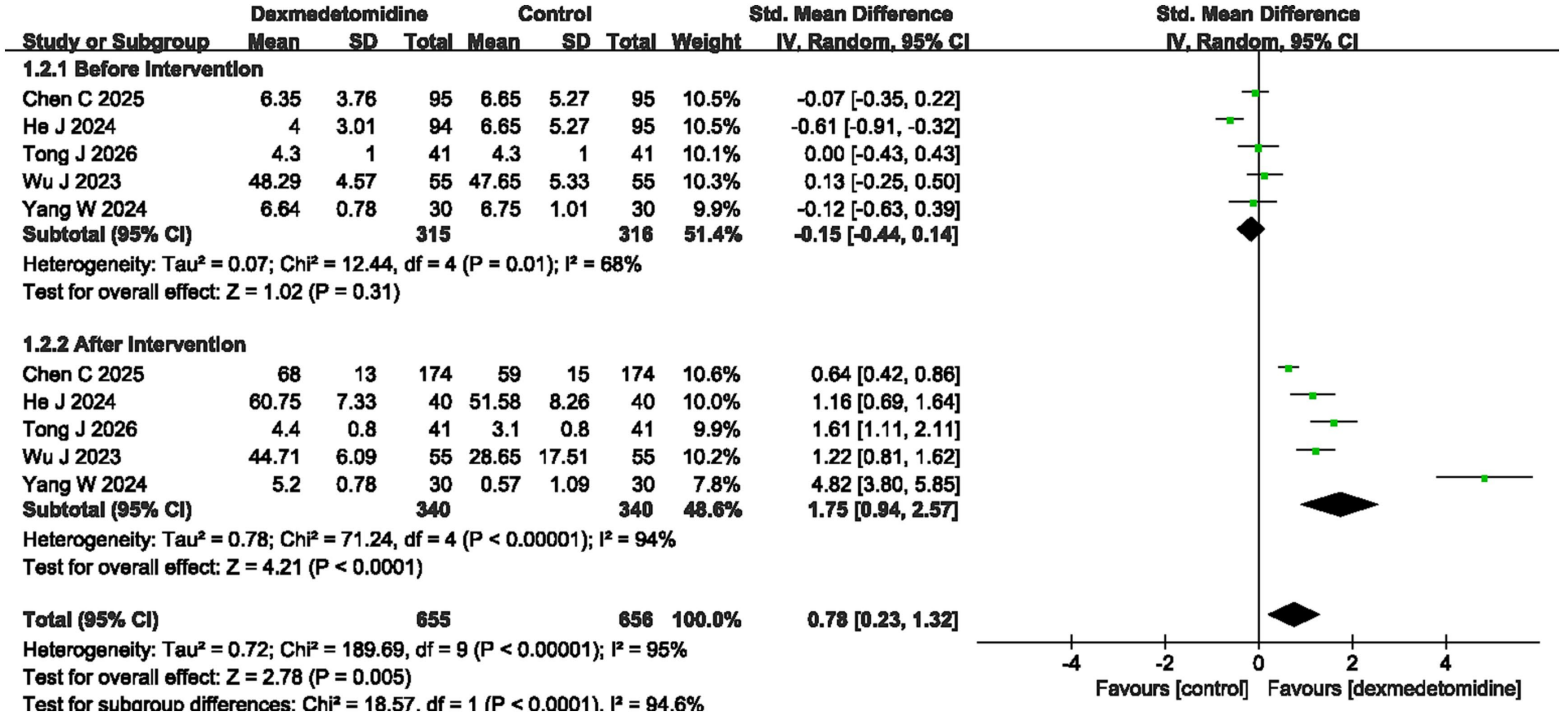
The effect of perioperative administration of intranasal dexmedetomidine on subjective sleep scores where a higher score means better sleep quality, including both baseline and postoperative results.

A total of 15 randomized controlled trials (16–18, 27–38) were included in the risk of bias assessment. As shown in Figure 2 and Figures 3, 4 studies did not report the method of allocation concealment. One study (17) used a blank control group, which may lead to unblinding of patients, so it was judged as high risk. Overall, the methodological quality of the included trials was considered acceptable. Publication bias was assessed only for the pooled analysis of postoperative subjective sleep quality, because the number of studies included in the remaining outcomes was fewer than 10. Visual inspection of the funnel plot showed a certain degree of asymmetry, suggesting the potential presence of publication bias (Supplementary Figure S1). Egger’s regression test further demonstrated significant small-study effects (*p* = 0.002). These findings indicated a possible risk of publication bias among the included studies. However, the relatively limited number of studies and the clinical heterogeneity among trials should also be considered when interpreting these results.

### 3.5 Subjective sleep quality score

The studies used different subjective tools to assess sleep quality, and the scoring rules were not the same. Therefore, we divided the results into two groups for the meta-analysis. In the first group, a lower score meant better sleep quality, including PSQI, NRS, and ISI. In the second group, a higher score meant better sleep quality, including SMHSQ, LSEQ and RCSQ. In 4 studies, the dexmedetomidine group had more than one dose, so we used labels such as DG1 and DG2 to show the different dose levels in the analysis.

**Figure 5:**
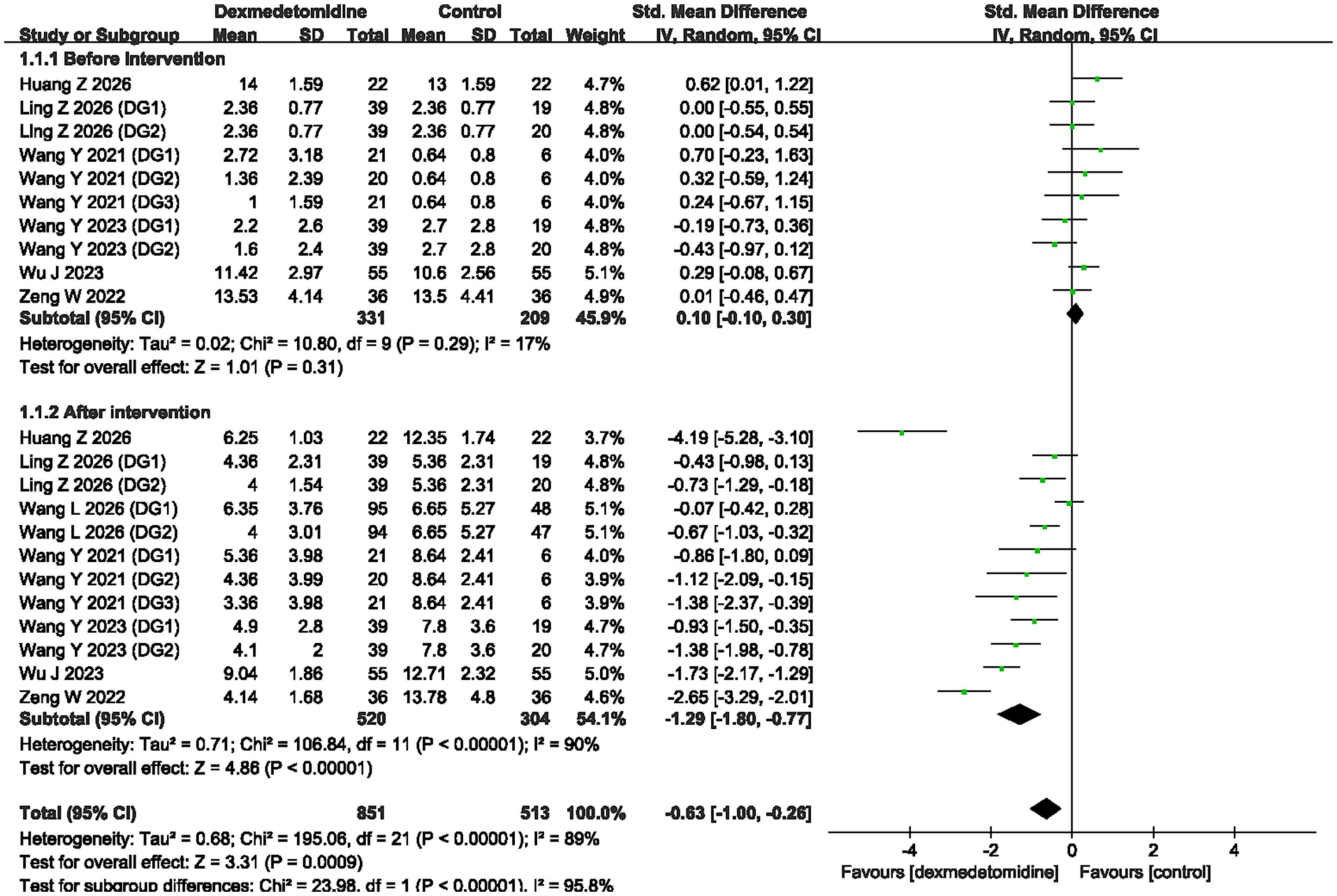
The effect of perioperative administration of intranasal dexmedetomidine on subjective sleep scores where a lower score means better sleep quality, including both baseline and postoperative results.

For the studies that used scores where a lower value meant better sleep quality, the results were analyzed in two time periods. Twelve datasets from 7 studies (16, 17, 29, 32, 34, 36, 37) were included. In the baseline period before surgery, the pooled result showed no significant difference between the intranasal dexmedetomidine group and the control group (SMD = 0.10, 95% CI [−0.10, 0.30], *I*^2^ = 17%, *p* = 0.31). In the period after the intervention, the pooled result showed that intranasal dexmedetomidine improved postoperative sleep quality compared with the control group (SMD = −1.29, 95% CI [−1.80, −0.77], *I*^2^ = 90%, *p* < 0.00001; Figure 5).

For the studies that used scores where a higher value meant better sleep quality, 5 studies (29, 30, 32, 35, 38) were included. In the baseline period before the intervention, the pooled analysis showed no significant difference between the intranasal dexmedetomidine group and the control group (SMD = −0.15, 95% CI [−0.44, 0.14], *I*^2^ = 68%, *p* = 0.31). After the intervention, the pooled result showed that intranasal dexmedetomidine improved postoperative sleep quality compared with the control group (SMD = 1.75, 95% CI [0.94, 2.57], *I*^2^ = 94%, *p* < 0.0001; Figure 4).

### 3.6 Subgroup analysis

Subgroup analyses were performed to explore the potential sources of heterogeneity in the pooled analysis of postoperative subjective sleep quality (Table 3).

**Table 3:** Effect sizes of the overall and subgroup analysis of the effects of intranasal dexmedetomidine on sleep quality.

Title	Number of trials	Number of participants	Statistical method	Effect size	Overall effect, *p* value	Heterogeneity *I*^2^ value
1. Overall effect	12	1,033	Std. Mean Difference (IV, Random, 95% CI)	−1.27 [−1.74, −0.80]	*p* < 0.00001	91%
2. Subgroup analysis by route of administration	12	Test for subgroup differences: *p* = 0.85				
2.1 Nasal spray	6	466	Std. Mean Difference (IV, Random, 95% CI)	−1.40 [−2.07, −0.73]	*p* < 0.0001	90%
2.2 Nasal drip	3	451	Std. Mean Difference (IV, Random, 95% CI)	−1.06 [−2.14, 0.01]	*p* = 0.05	96%
2.3 Nasal packing	3	116	Std. Mean Difference (IV, Random, 95% CI)	−1.24 [−1.64, −0.83]	*p* < 0.00001	0%
3. Subgroup analysis by timing of administration	12	Test for subgroup differences: *p* = 0.007				
3.1 Before bedtime	7	538	Std. Mean Difference (IV, Random, 95% CI)	−1.59 [−2.28, −0.91]	*p* < 0.00001	92%
3.2 Before induction	2	379	Std. Mean Difference (IV, Random, 95% CI)	−0.34 [−0.88, 0.20]	*p* = 0.22	86%
3.3 Others	3	116	Std. Mean Difference (IV, Random, 95% CI)	−1.24 [−1.64, −0.83]	*p* < 0.00001	0%
4. Subgroup analysis by duration of administration	12	Test for subgroup differences: *p* = 0.37				
4.1 Once	9	767	Std. Mean Difference (IV, Random, 95% CI)	−1.40 [−2.00, −0.80]	*p* < 0.00001	92%
4.2 Twice or more times	3	266	Std. Mean Difference (IV, Random, 95% CI)	−0.95 [−1.74, −0.16]	*p* = 0.02	89%
5. Subgroup analysis by age	12	Test for subgroup differences: *p* = 0.12				
5.1 <60 years	10	879	Std. Mean Difference (IV, Random, 95% CI)	−0.99 [−1.40, −0.58]	*p* < 0.00001	87%
5.2 ≥60 years	2	154	Std. Mean Difference (IV, Random, 95% CI)	−2.91 [−5.31, −0.50]	*p* = 0.02	94%
6. Subgroup analysis by sex	12	Test for subgroup differences: *p* = 0.003				
6.1 Female-dominant	2	379	Std. Mean Difference (IV, Random, 95% CI)	−0.34 [−0.88, 0.20]	*p* = 0.22	86%
6.1 Mixed population	10	654	Std. Mean Difference (IV, Random, 95% CI)	−0.39 [−0.67, −0.10]	*p* < 0.00001	88%
7. Subgroup analysis by surgical type	12	Test for subgroup differences: *p* = 0.02				
7.1 Abdominal surgery	2	379	Std. Mean Difference (IV, Random, 95% CI)	−0.34 [−0.88, 0.20]	*p* = 0.22	86%
7.2 Orthopedic surgery	2	154	Std. Mean Difference (IV, Random, 95% CI)	−2.91 [−5.31, −0.50]	*p* = 0.02	94%
7.3 Others	8	500	Std. Mean Difference (IV, Random, 95% CI)	−1.18 [−1.63, −0.73]	*p* < 0.00001	81%
8. Subgroup based on anesthesia type	11	Test for subgroup differences: *p* = 0.58				
8.1 General anesthesia	8	538	Std. Mean Difference (IV, Random, 95% CI)	−1.08 [−1.41, −0.74]	*p* < 0.00001	69%
8.2 Others	3	423	Std. Mean Difference (IV, Random, 95% CI)	−1.42 [−2.61, −0.23]	*p* = 0.02	96%
9. Subgroup based on evaluation scale	12	Test for subgroup differences: *p* = 0.94				
9.1 PSQI	9	805	Std. Mean Difference (IV, Random, 95% CI)	−1.29 [−1.82, −0.75]	*p* < 0.00001	91%
9.2 Other scales	3	228	Std. Mean Difference (IV, Random, 95% CI)	−1.23 [−2.43, −0.03]	*p* = 0.04	94%

NA: not applicable.

In the subgroup analysis based on the route of administration, significant improvements in postoperative sleep quality were observed in the nasal spray subgroup (SMD = −1.40, 95% CI [−2.07, −0.73], *p* < 0.0001) and the nasal packing subgroup (SMD = −1.24, 95% CI [−1.64, −0.83], *p* < 0.00001), whereas the nasal drip subgroup showed a borderline effect (SMD = −1.06, 95% CI [−2.14, 0.01], *p* = 0.05). However, no significant subgroup difference was found among the different administration routes (*p* = 0.85).

Subgroup analysis according to the timing of administration showed a significant subgroup difference (*p* = 0.007). Intranasal dexmedetomidine administered before bedtime significantly improved postoperative sleep quality (SMD = −1.59, 95% CI [−2.28, −0.91], *p* < 0.00001), whereas administration before anesthesia induction did not show a significant benefit (SMD = −0.34, 95% CI [−0.88, 0.20], *p* = 0.22). Studies classified as “others” also demonstrated significant improvement (SMD = −1.24, 95% CI [−1.64, −0.83], *p* < 0.00001).

In the subgroup analysis based on the duration of administration, both single-dose administration (SMD = −1.40, 95% CI [−2.00, −0.80], < 0.00001) and repeated administration (SMD = −0.95, 95% CI [−1.74, −0.16], *p* = 0.02) were associated with improved postoperative sleep quality, with no significant subgroup difference between them (*p* = 0.37).

Subgroup analysis by age demonstrated significant improvements in both patients younger than 60 years (SMD = −0.99, 95% CI [−1.40, −0.58], *p* < 0.00001) and those aged 60 years or older (SMD = −2.91, 95% CI [−5.31, −0.50], *p* = 0.02), without a statistically significant subgroup difference (*p* = 0.12).

In the subgroup analysis based on sex, a significant subgroup difference was observed (*p* = 0.003). The mixed-population subgroup showed a significant improvement in postoperative sleep quality (SMD = −0.39, 95% CI [−0.67, −0.10], *p* < 0.00001), whereas the female-dominant subgroup did not demonstrate a statistically significant effect (SMD = −0.34, 95% CI [−0.88, 0.20], *p* = 0.22).

Subgroup analysis according to surgical type also revealed significant subgroup differences (*p* = 0.02). Significant improvements in postoperative sleep quality were observed in orthopedic surgery (SMD = −2.91, 95% CI [−5.31, −0.50], *p* = 0.02) and other surgery types (SMD = −1.18, 95% CI [−1.63, −0.73], *p* < 0.00001), whereas no significant effect was found in abdominal surgery (SMD = −0.34, 95% CI [−0.88, 0.20], *p* = 0.22).

In the subgroup analysis based on anesthesia type, significant improvements in postoperative sleep quality were observed in both the general anesthesia subgroup (SMD = −1.08, 95% CI [−1.41, −0.74], *p* < 0.00001) and the other anesthesia subgroup (SMD = −1.42, 95% CI [−2.61, −0.23], *p* = 0.02), with no significant subgroup difference between them (*p* = 0.58).

Subgroup analysis according to evaluation scale showed that both the PSQI subgroup (SMD = −1.29, 95% CI [−1.82, −0.75], *p* < 0.00001) and the other scales subgroup (SMD = −1.23, 95% CI [−2.43, −0.03], *p* = 0.04) demonstrated significant improvements in postoperative sleep quality, without a significant subgroup difference (*p* = 0.94).

Overall, the subgroup analyses suggested that the timing of administration, sex distribution, and surgical type might be important contributors to the observed heterogeneity.

### 3.7 Sensitivity analyses

Sensitivity analyses were performed by excluding studies assessed as having a high risk of bias. After exclusion of one study using blank control group, the pooled analysis of postoperative sleep quality remained statistically significant (SMD = −1.32 95% CI [−1.94, −0.70], *p* < 0.0001), and the direction of the effect did not change. These findings suggested that the results of the present meta-analysis were robust and stable.

### 3.8 Objective sleep quality score

**Figure 6:**
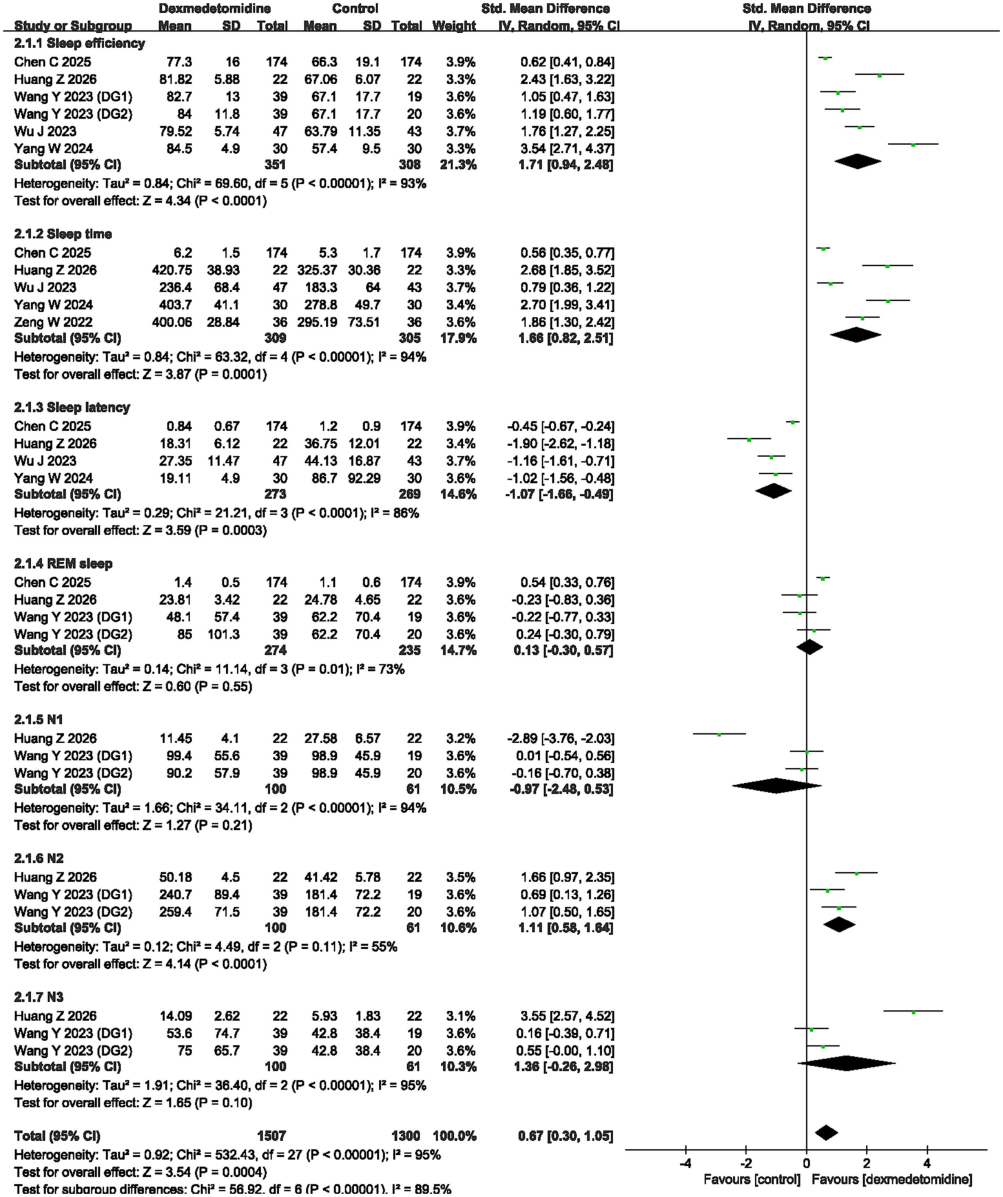
The effect of perioperative administration of intranasal dexmedetomidine on objective sleep outcome.

Six studies (17, 29, 32, 33, 36, 38) reported objective sleep quality outcomes, including sleep efficiency, total sleep time, REM sleep, and sleep stage parameters (N1, N2, and N3 sleep). The pooled result showed that intranasal dexmedetomidine improved sleep efficiency compared with the control group (SMD = 1.71, 95% CI [0.94, 2.48], *I*^2^ = 93%, *p* < 0.0001). Intranasal dexmedetomidine also significantly increased sleep time (SMD = 1.66, 95% CI [0.82, 2.51], *I*^2^ = 94%, *p* = 0.0001) and reduced sleep latency (SMD = −1.07, 95% CI [−1.66, −0.49], *I*^2^ = 86%, *p* = 0.0003). For REM sleep duration, no significant difference was observed between the dexmedetomidine and control groups (SMD = 0.13, 95% CI [−0.30, 0.57], *I*^2^ = 73%, *p* = 0.55). Similarly, no significant difference was found for N1 (SMD = −0.97, 95% CI [−2.48, 0.53], *I*^2^ = 94%, *p* = 0.21) and N3 sleep (SMD = 1.36, 95% CI [−0.26, 2.98], *I*^2^ = 95%, *p* = 0.10). However, intranasal dexmedetomidine significantly increased N2 sleep duration (SMD = 1.11, 95% CI [0.58, 1.64], *I*^2^ = 55%, *p* < 0.0001; Figure 6).

### 3.9 Incidence of sleep disturbance

**Figure 7:**
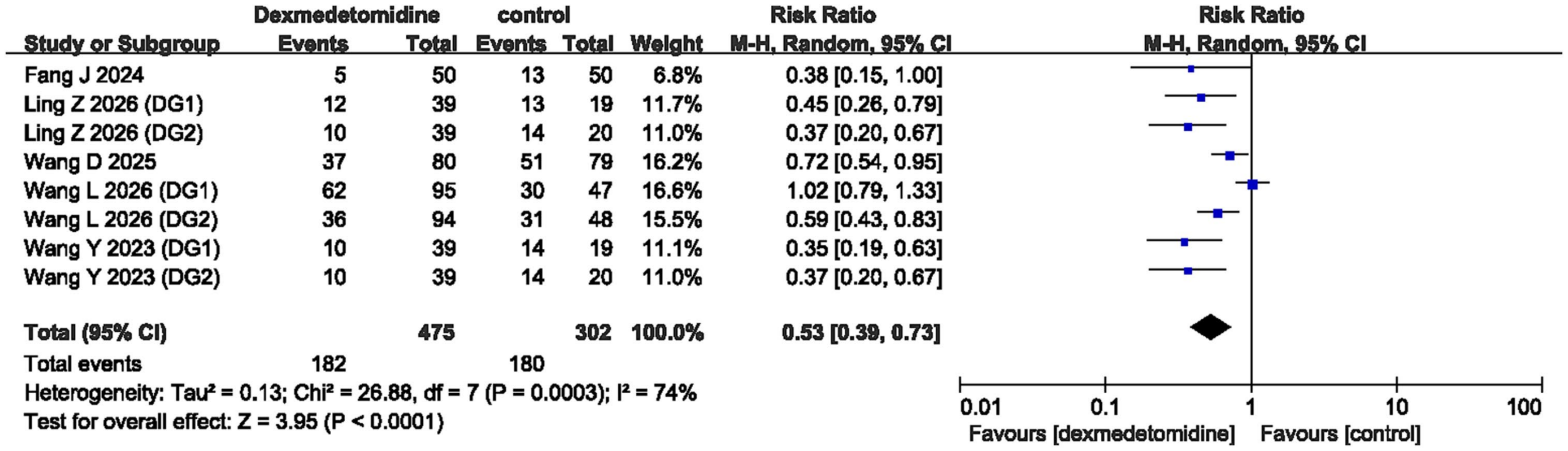
The effect of perioperative administration of intranasal dexmedetomidine on the incidence of postoperative sleep disturbance.

Five studies (17, 27, 28, 34, 37) with a total of 8 intervention groups reported the incidence of sleep disturbance. The pooled analysis showed that intranasal dexmedetomidine reduced the risk of postoperative sleep disturbance compared with the control group (RR = 0.53, 95% CI [0.39, 0.73], *I*^2^ = 74%, *p* < 0.0001; Figure 7).

### 3.10 Adverse events

**Figure 8:**
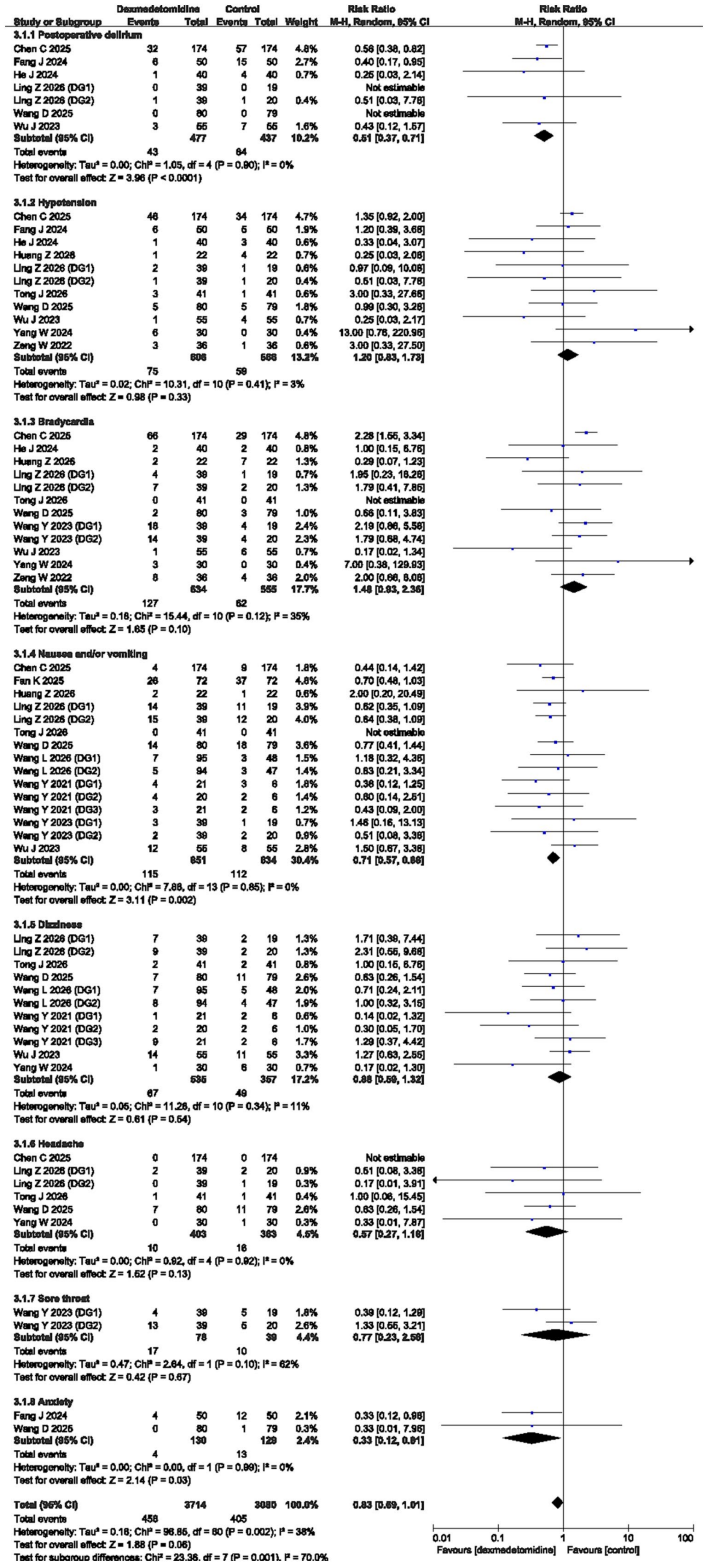
Forest plot of adverse events.

Several studies reported different adverse events, including postoperative delirium, hypotension, bradycardia, nausea and vomiting, dizziness, headache, sore throat, and anxiety. The pooled analysis showed that intranasal dexmedetomidine significantly reduced the risk of postoperative delirium compared with the control group (RR = 0.51, 95% CI [0.37, 0.71], *I*^2^ = 0%, *p* < 0.0001). There was no significant difference between the two groups in the risk of hypotension (RR = 1.20, 95% CI [0.83, 1.73], *I*^2^ = 3%, *p* = 0.33) and bradycardia (RR = 1.48, 95% CI [0.93, 2.36], *I*^2^ = 35%, *p* = 0.10). Intranasal dexmedetomidine significantly reduced the incidence of nausea and/or vomiting compared with the control group (RR = 0.71, 95% CI [0.57, 0.88], *I*^2^ = 0%, *p* = 0.002). No significant differences were observed between the two groups in the incidence of dizziness (RR = 0.88, 95% CI [0.59, 1.32], *I*^2^ = 11%, *p* = 0.54), headache (RR = 0.57, 95% CI [0.27, 1.18], *I*^2^ = 0%, *p* = 0.13), or sore throat (RR = 0.77, 95% CI [0.23, 2.56], *I*^2^ = 62%, *p* = 0.67). For anxiety, intranasal dexmedetomidine significantly reduced the risk compared with the control group (RR = 0.33, 95% CI [0.12, 0.91], *I*^2^ = 0%, *p* = 0.03; Figure 8).

### 3.11 GRADE assessment of the certainty of evidence

The GRADE assessment showed that the certainty of evidence was low or very low for most outcomes, mainly because of risk of bias, substantial heterogeneity, indirectness related to heterogeneous intervention regimens, imprecision in several outcomes, and possible publication bias for subjective sleep quality (Supplementary Table S3).

## 4 Discussion

The key findings of this review indicated that intranasal dexmedetomidine improved both subjective and objective postoperative sleep quality. Patients receiving intranasal dexmedetomidine showed better postoperative subjective sleep scores, higher sleep efficiency and total sleep time, shorter sleep latency, and increased N2 sleep duration. Subgroup analyses suggested that the timing of administration, sex distribution, and surgical type might be important sources of heterogeneity in subjective sleep outcomes. In addition, intranasal dexmedetomidine reduced the incidence of postoperative sleep disturbance, delirium, nausea and/or vomiting, and anxiety. Together, these findings indicate that intranasal dexmedetomidine could serve as a useful and safe approach for enhancing sleep quality after surgery.

The findings of our review were generally consistent with previous meta-analyses that evaluated intravenous dexmedetomidine. Wang and colleagues (9) pooled 29 randomized trials and found that perioperative intravenous dexmedetomidine improved subjective sleep scores on the first postoperative night and increased sleep efficiency, although the evidence quality was low and heterogeneity was high. Liu and colleagues (7) focused on polysomnography data and also reported that dexmedetomidine increased sleep efficiency and total sleep time, reduced arousal index and stage N1 sleep, and increased stage N2 sleep, with no clear effect on stage N3 or REM sleep. Our results with intranasal dexmedetomidine show a similar pattern, with better subjective sleep scores, higher sleep efficiency and longer total sleep time, and no significant change in REM sleep. However, unlike the intravenous studies where bradycardia and hypotension were more frequent, our pooled analysis did not show a clear increase in cardiovascular adverse events. These comparisons suggest that intranasal dexmedetomidine may provide sleep benefits that are comparable to intravenous use, while offering a less invasive route of administration.

Although both intranasal and intravenous administration can improve sleep quality, studies that compared the two routes directly were still limited. Niu J et al. (31) compared intranasal dexmedetomidine with intravenous or intratracheal routes instead of saline or blank control, and the doses and pharmacokinetics were very different from the other trials, we did not include it in the meta-analysis but discussed it narratively. In this study, older adults scheduled for elective spine surgery were randomly allocated to three groups that received dexmedetomidine by different routes: intravenous infusion at 0.6 μg/kg, intranasal drops at 1 μg/kg, or intratracheal administration at 0.6 μg/kg mixed with ropivacaine. On the second postoperative morning, the group that received the intravenous dose showed the lowest PSQI score, indicating better sleep than both the intranasal and intratracheal groups.

Several mechanisms may explain the different effects of intranasal and intravenous dexmedetomidine on sleep. First, pharmacokinetic studies showed that intranasal dexmedetomidine had a bioavailability of about 40%, with slower absorption and later peak plasma concentration than intravenous infusion, but a similar duration of sedation. This produced a more gradual onset and smoother time–concentration curve, which may be closer to natural sleep and may reduce haemodynamic fluctuations seen with rapid intravenous dosing (12). Second, intranasal administration can deliver drug not only through the systemic circulation but also directly along the nose–brain pathway via the olfactory and trigeminal nerves, partly bypassing the blood–brain barrier. This may enhance central nervous system targeting while limiting systemic exposure and could help to stabilize sleep without increasing peripheral side effects (39). Third, polysomnography studies of intravenous dexmedetomidine showed that it promoted non-REM sleep, increased sleep efficiency and N2 or slow-wave sleep, but higher systemic doses were often limited by bradycardia and hypotension (40). Intranasal dosing may achieve a similar modulation of sleep architecture at lower effective plasma concentrations, which could explain why our review found improved sleep quality with intranasal dexmedetomidine while cardiovascular adverse events did not increase.

In addition to intravenous and intranasal administration, several recent studies explored other routes of dexmedetomidine for sleep regulation. A pharmacokinetic and pharmacodynamic trial in volunteers showed that sublingual and especially buccal oromucosal dexmedetomidine shortened sleep latency, increased NREM sleep, and raised slow wave activity in poor sleepers, without clear next morning impairment (41). In women undergoing cesarean delivery, continuous low dose epidural dexmedetomidine during and after surgery reduced the incidence of postpartum sleep disturbance and improved subjective sleep scores compared with epidural analgesia alone (42). A randomized crossover study in healthy adults found that an oral capsule formulation of dexmedetomidine increased non rapid eye movement stage 2 sleep and decreased rapid eye movement sleep on polysomnography, although it did not improve sleep dependent motor memory (8). Together, these findings suggested that multiple non intravenous routes of dexmedetomidine, including oromucosal, epidural, and oral administration, also had important effects on sleep architecture and perceived sleep quality.

In our review, intranasal dexmedetomidine was given in several different ways, including nasal spray, drops from a syringe and nasal packing, with wide variation in dose and timing. Pharmacokinetic studies (12) showed that both nasal spray and drops produced adequate plasma concentrations, but nasal spray seemed to have slightly higher bioavailability, which might have led to faster and more stable sedation in some settings (43). A recent randomized trial (44) in children found that ready to use nasal spray and traditional drops achieved similar sedation, while the spray was more acceptable and easier to use, which supported the idea that formulation mainly influenced comfort and cooperation rather than basic sedative effect. Other studies suggested that within a dose range of about 1 to 2 micrograms per kilogram, higher doses did not always shorten onset time or markedly prolong sedation, which indicated a wide therapeutic window (45, 46). For postoperative sleep, one trial that compared 1.0 and 1.5 micrograms per kilogram nasal spray reported better sleep efficiency and longer N2 and even N3 sleep in the higher dose group, which suggested a possible dose response effect on sleep architecture (17). In our included studies, some protocols used a fixed 50 microgram spray about half an hour before patients went to sleep on the first night after surgery, whereas others used weight-based doses given before anesthesia or on several perioperative nights, yet most regimens improved sleep quality. Taken together, these data indicated that different intranasal formulations, doses and timing might influence the speed of onset, patient acceptance and the degree of change in sleep structure, and they likely contributed to the heterogeneity in our meta-analysis, but all fell within an effective range for improving perioperative sleep.

This review had several strengths. We focused specifically on intranasal dexmedetomidine, which was a newly developing route and had not been evaluated in previous meta-analyses. This helped fill an important knowledge gap and provided new evidence for clinical practice. Additionally, the review included both subjective and objective sleep outcomes, which allowed a more complete understanding of the effect on sleep. The use of different questionnaires and sleep monitoring devices made the analysis more comprehensive. Furthermore, every study included in the review used a randomized controlled design, which increased the reliability of the results.

Several limitations need to be recognized. First, the total number of eligible studies was small, and all of them were carried out in China, which may limit external validity because sleep patterns, perioperative management, and healthcare systems may differ across regions. Second, substantial heterogeneity was observed across several pooled outcomes. This heterogeneity was likely attributable to timing of administration, sex distribution, and surgical type. Therefore, the certainty and generalizability of the evidence remain limited. Third, the studies used different tools to measure sleep quality, and the lack of a unified evaluation method made it difficult to compare outcomes directly. Fourth, publication bias was assessed only for the main analysis of postoperative subjective sleep quality, in which funnel plot asymmetry and Egger’s test suggested possible small-study effects. For most secondary outcomes, publication bias could not be formally assessed because fewer than 10 studies were available. Therefore, the possibility of publication bias cannot be excluded. Finally, the optimal intranasal dexmedetomidine dose for improving postoperative sleep quality remains uncertain and should be evaluated in future head-to-head randomized trials using standardized protocols.

## 5 Conclusion

In conclusion, the findings of this review showed that intranasal dexmedetomidine improved both subjective and objective sleep quality after surgery. It reduced the likelihood of sleep disturbance and showed a favorable safety profile. These findings indicate that intranasal dexmedetomidine could offer a practical and convenient approach to enhancing postoperative sleep in adult surgical patients. However, the available evidence remains limited, and additional large, well-designed clinical trials are required to verify these findings and to establish the most appropriate intranasal dose and timing.

## Data Availability

The original contributions presented in the study are included in the article/Supplementary material, further inquiries can be directed to the corresponding author.
