# Supplementary figures and images for: Effects of intranasal dexmedetomidine on postoperative sleep quality: a systematic review and meta-analysis of randomized controlled trials
Source: Front Med (Lausanne). 2026 Jul 10;13:1890318. doi: 10.3389/fmed.2026.1890318 (PMC13395764; doi:10.3389/fmed.2026.1890318)

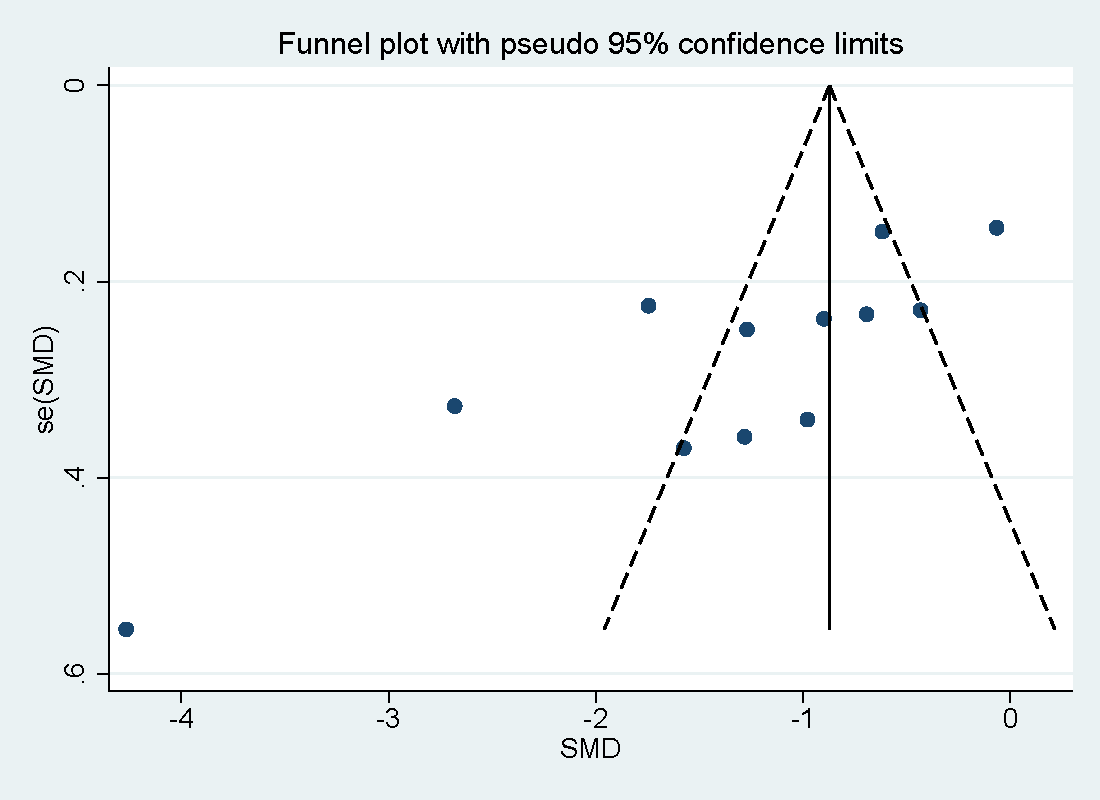

Supplement: Supplementary file 1 [file Image_1.TIF]
