# Supplementary material for: Effects of intranasal dexmedetomidine on postoperative sleep quality: a systematic review and meta-analysis of randomized controlled trials
Source: Front Med (Lausanne). 2026 Jul 10;13:1890318. doi: 10.3389/fmed.2026.1890318 (PMC13395764; doi:10.3389/fmed.2026.1890318)
Supplement: Supplementary file 3 [file Table_2.DOCX]

**Table S2**

Literature search strategy.

| **Electronic database** | **Search terms** | **Number of articles** |
| --- | --- | --- |
| PubMed | #1 Search: ("Dexmedetomidine"[Mesh]) OR (((((((Dexmedetomidine[Title/Abstract]) OR (Dexmedetomidine Hydrochloride[Title/Abstract])) OR (Hydrochloride, Dexmedetomidine[Title/Abstract])) OR (MPV-1440[Title/Abstract])) OR (MPV1440[Title/Abstract])) OR (MPV 1440[Title/Abstract])) OR (Precedex[Title/Abstract]))  #2 Search: ("Administration, Intranasal"[Mesh]) OR (((Spray[Title/Abstract]) OR (Intranasal[Title/Abstract])) OR (Nasal[Title/Abstract]))  #3 Search: ("Sleep"[Mesh]) OR ((((((((((Sleep[Title/Abstract]) OR (Sleep Quality[Title/Abstract])) OR (Qualities, Sleep[Title/Abstract])) OR (Quality, Sleep[Title/Abstract])) OR (Sleep Qualities[Title/Abstract])) OR (Insomnia[Title/Abstract])) OR (Sleep Disorders[Title/Abstract])) OR (Sleep Disturbance[Title/Abstract])) OR (Sleeplessness[Title/Abstract])) OR (Rest activity[Title/Abstract]))  #1 And #2 And #3 | 65 |
| Embase | #1 'dexmedetomidine'/exp  #2 'dexmedetomidine':ab,ti OR 'dexmedetomidine hydrochloride':ab,ti OR 'hydrochloride, dexmedetomidine':ab,ti OR 'mpv-1440':ab,ti OR 'mpv1440':ab,ti OR 'mpv 1440':ab,ti OR 'precedex':ab,ti  #3 #1 OR #2  #4 'intranasal drug administrations'/exp  #5 'spray':ab,ti OR 'intranasal':ab,ti OR 'nasal':ab,ti  #6 #4 OR #5  #7 'sleep'/exp  #8 'sleep':ab,ti OR 'sleep quality':ab,ti OR 'qualities, sleep':ab,ti OR 'insomnia':ab,ti OR 'sleep disorders':ab,ti OR 'sleep disturbance':ab,ti OR 'sleeplessness':ab,ti OR 'rest activity':ab,ti  #9 #7 OR #8  #10 'randomized controlled trial'/exp  #11 #3 AND #6 AND #9 AND #10 | 58 |
| Cochrane Library | #1 MeSH descriptor:[Dexmedetomidine] explode all trees  #2 (Dexmedetomidine): ti,ab,kw OR (Dexmedetomidine Hydrochloride): ti,ab,kw OR (Hydrochloride, Dexmedetomidine): ti,ab,kw OR (MPV-1440): ti,ab,kw OR (MPV1440): ti,ab,kw OR (MPV 1440): ti,ab,kw OR (Precedex): ti,ab,kw  #3 #1 OR #2  #4 MeSH descriptor:[Administration, Intranasal] explode all trees  #5 (Spray): ti,ab,kw OR (Intranasal): ti,ab,kw OR (Nasal): ti,ab,kw  #6 #4 OR #5  #7 MeSH descriptor:[Sleep] explode all trees  #8 (Sleep): ti,ab,kw OR (Sleep Quality): ti,ab,kw OR (Qualities, Sleep): ti,ab,kw OR (Quality, Sleep): ti,ab,kw OR (Sleep Qualities): ti,ab,kw OR (Insomnia): ti,ab,kw OR (Sleep Disorders): ti,ab,kw OR (Sleep Disturbance): ti,ab,kw OR (Sleeplessness): ti,ab,kw OR (Rest activity): ti,ab,kw  #9 #7 OR #8  #10 #3 AND #6 AND #9 | 143 |
