# Supplementary material for: Effects of intranasal dexmedetomidine on postoperative sleep quality: a systematic review and meta-analysis of randomized controlled trials
Source: Front Med (Lausanne). 2026 Jul 10;13:1890318. doi: 10.3389/fmed.2026.1890318 (PMC13395764; doi:10.3389/fmed.2026.1890318)
Supplement: Supplementary file 4 [file Table_3.DOCX]

**Table S3**

GRADE assessment of the certainty of evidence.

| **Outcome** | **No. of studies/ participants** | **Effect estimate** | **Heterogeneity** | **Certainty of evidence** | **Main reasons for downgrading** |
| --- | --- | --- | --- | --- | --- |
| Subjective sleep quality, lower score indicating better sleep | 7 studies/ 824 | SMD = −1.29, 95% CI [−1.80, −0.77] | I^2^ = 90% | ⬤◯◯◯ Very low | Possible publication bias; differences in scales, doses, timing, and comparators; high heterogeneity (I^2^>90%) |
| Subjective sleep quality, higher score indicating better sleep | 5 studies/ 680 | SMD = 1.75, 95% CI [0.94, 2.57] | I^2^ = 94% | ⬤◯◯◯ Very low | Indirectness due to different subjective sleep scales, doses, timing, and perioperative settings; high heterogeneity (I^2^>90%) |
| Incidence of postoperative sleep disturbance | 5 studies/ 777 | RR = 0.53, 95% CI [0.39, 0.73] | I^2^ = 74% | ⬤⬤◯◯ Low | Indirectness due to different definitions of sleep disturbance |
| Sleep efficiency | \| 5 studies/ 659 \| \| --- \| | SMD = 1.71, 95% CI [0.94, 2.48] | I^2^ = 93% | ⬤⬤◯◯ Low | High heterogeneity (I^2^>90%) |
| Nausea and/or vomiting | 8 studies/ 1485 | RR = 0.71, 95% CI [0.57, 0.88] | I^2^ = 0% | ⬤⬤⬤◯ Moderate | China-only studies |

**Abbreviations:** GRADE: Grading of Recommendations Assessment, Development and Evaluation; SMD: standardized mean difference; RR: risk ratio; CI: confidence interval.
